# lncRNA FAM230B is highly expressed in colorectal cancer and suppresses the maturation of miR-1182 to increase cell proliferation

**DOI:** 10.1515/med-2022-0500

**Published:** 2022-10-03

**Authors:** Ni Li, Chuane Zhou, Fan Yang

**Affiliations:** Department of Oncology, Chongqing Jiulongpo District People’s Hospital, Yangjiaping, Jiulongpo District, Chongqing City, 400050, P.R. China; Department of Oncology, Jianshi County People’s Hospital, Enshi Prefecture, Hubei Province, Jianshi County, Enshi Prefecture, Hubei Province, 445300, P.R. China; Department of Oncology, The Second Affiliated Hospital of Army Military Medical University, 20-8, Building C, Buke Mansion, Fengtian Road, Shapingba, Chongqing City, 400050, P.R. China

**Keywords:** colorectal cancer, FAM230B, miR-1182, maturation, proliferation

## Abstract

Long non-coding RNA FAM230B and microRNA (miR-1182) have been characterized as critical players in cancer biology, while their roles in colorectal cancer (CRC) are unclear. We predicted that they could interact with each other and therefore explored the interaction between them in CRC. CRC and paired non-tumor tissue samples were collected from 60 CRC patients, and the expression of FAM230B and miR-1182 (premature and mature) in these samples was analyzed with RT-qPCR. The direct interaction between FAM230B and premature miR-1182 was analyzed with RNA-RNA pull-down assay, and the subcellular location of FAM230B was detected with subcellular fractionation assay. The interaction between FAM230B and miR-1182 was explored with overexpression assay, and their roles in regulating CRC cell proliferation, viability, and colony formation were assessed by BrdU assay, MTT assay, and colony formation assay, respectively. We found that FAM230B and premature miR-1182 were highly upregulated in CRC, while mature miR-1182 was downregulated in CRC. FAM230B was detected in both nucleus and cytoplasm, and it directly interacted with miR-1182. FAM230B overexpression increased the expression levels of premature miR-1182 but decreased the expression levels of mature miR-1182 in CRC cells. FAM230B promoted CRC cell proliferation, increased cell viability, accelerated colony formation, and suppressed the role of miR-1182 in inhibiting CRC cell proliferation. In conclusion, FAM230B is upregulated in CRC and it suppresses the maturation of miR-1182 to promote tumor growth.

## 1 Introduction

As a type of solid malignant tumor that develops from cells in rectum and/or colon, colorectal cancer (CRC), a common malignancy in clinical practices, affects about 1 out of 25 women and 1 out of 23 men during their lifetime [1,2]. CRC mostly affects patients older than 60 years old, while in recent years CRC cases are increasing among young adults [3,4]. CRC patients diagnosed with tumor localized to the bowel can usually be cured by surgical resection [5], while often there is no cure for metastatic CRC [6]. Unfortunately, about 60% of CRC patients are diagnosed with tumor metastasis outside of colon or rectum [7], leading to a low overall 5-year survival rate below 20% [8].

Although a small portion of patients with tumor metastasis limited to distant organs, such as the lungs and liver, can be cured by surgery, most metastatic CRC patients are treated with chemotherapy but outcomes are generally poor, mainly owing to the development of side effects [9,10]. Targeted therapy is an emerging approach to treat cancers [11]. However, this novel approach is still under research and the identification of more targets are still needed [12,13,14]. Long non-coding RNAs (lncRNAs) and microRNAs (miRNAs) do not have protein-coding capacity, but indirectly affect the synthesis and accumulation of proteins, suggesting that they are promising targets to treat cancers [12,13,14]. However, the roles of most lncRNAs in cancers are unclear. lncRNA FAM230B and miR-1182 have been characterized as critical players in cancer biology [15,16,17], while their role in CRC is unclear. We predicted that they could interact with each other. This study was therefore carried out to explore the interaction between them in CRC.

## 2 Materials and methods

### 2.1 Patients and tissue specimens

The present study enrolled a total of 64 CRC patients (30 females and 34 males, mean age 55.5 ± 7.8 years old) at the Second Affiliated Hospital of Army Military Medical University from May 2019 to May 2021 to serve as the research subjects. All CRC patients were newly diagnosed cases and other clinical disorders were excluded. Patients with blood relationship were excluded. The 64 patients included 28 cases at stage I/II and 36 cases at stage III/IV. CRC and paired non-tumor tissue samples were obtained from each participant either by performing biopsies or dissecting resected primary tumors.


**Ethical approval and consent to participate:** All patients signed the written informed consent. All procedures were approved by the Ethics Committee of the Second Affiliated Hospital of Army Military Medical University. Procedures operated in this research were completed in keeping with the standards set out in the Announcement of Helsinki and laboratory guidelines of research in China.

### 2.2 Cell culture

McCoy’s 5A medium (Gibco) supplemented with FBS (10%) and penicillin–streptomycin (1%) was used to maintain human CRC cell lines SW480 and HCT116, which were used to perform *in vitro* cell experiments in this study. No mycoplasma was detected in both the cell lines. Cell culture was performed in an incubator with 5% CO_2_ (v/v) and temperature and humidity were set to 37°C and 95%, respectively.

### 2.3 Cell transfection

Cells were transfected withFAM230B vector (pCMV3, Addgene) and/or miR-1182 mimic (Invitrogen) to overexpress FAM230B and/or miR-1182. Cells used for transfection were seeded onto a 6-well plate and cultivated overnight prior to the incubation with transfection mixture, which was composed of Lipofectamine 2000 (Invitrogen) and vector and/or miR-1182 mimic. Incubation with transfection mixture was performed for 6 h. After that, cells were transferred to fresh medium in a 6-well plate to reduce cytotoxicity. Each transfection included 10^7^ cells, and the dose of miR-1182 and vector was 50 and 10 mM, respectively.

### 2.4 RNA sample preparation

EasyPure^®^ RNA Purification Kit was used to isolate total RNAs from all samples following the manufacturer’s instructions. Briefly, columns were used for RNA binding through centrifugation, followed by washing using washing buffer. After that, RNA elution was performed using RNase-free water. RNA samples were first digested with DNase I at 37°C for 2 h to completely remove genomic DNA, followed by measuring the RNA concentrations using Nanodrop 2000. Bioanalyzer (2100) was used to determine RNA integrity.

### 2.5 RT-qPCR

Nuclease-free water was added to RNA samples to adjust RNA concentrations to about 2,000 ng/μL. With 2 μL RNA sample as template, cDNA samples were synthesized using PrimeScript™ Reverse (Takara). The housekeeping gene GAPDH was amplified to test the quality of cDNA samples. With cDNA samples as template, qPCR was performed to determine the expression of FAM230B and miR-1182 (mature and premature) with 18S rRNA as the internal control. Relative gene expression levels were calculated by normalizing to 18S rRNA using the 2^−ΔΔCT^ method.

### 2.6 Cellular fractionation assay

Nuclear and Cytoplasmic Protein Extraction Kit (Beyotime) was used to prepare both cytoplasmic and nuclear fractions from 10^7^ cells. Briefly, cells were harvested and dissolved in cell lysis buffer, after incubation on ice for 20 min, cell lysates were centrifuged in cold room at 1,200*g* for 15 min to separate nuclear fraction and cytoplasmic fraction. The supernatant, which was the cytoplasmic fraction, was transferred to a nuclease-free tube, and the cell pellet, which was the nuclear fraction, was further incubated with cell lysis buffer on ice for further 20 min. After that, nuclear fraction was collected after centrifugation in cold room at 1,200*g* for 10 min. RNA samples were then isolated and purified, which were subjected to RT-PCR to determine the expression of FAM230B.

### 2.7 RNA–RNA pulldown assay

FAM230B and negative control RNAs used in this assay were *in vitro* transcripts prepared using T3 RNA Polymerase (NEB). The 3′ end of both RNAs were biotinylated using Pierce™ RNA 3′ End Biotinylation Kit (Thermal Fisher). These two labeled RNAs (Bio-xx and Bio-NC) were transfected into cells using the method mentioned above. Cells were collected 48 h later and cell lysates were prepared by incubating the cells with cell lysis buffer on ice for 20 min. After that, Dynabeads M-280 Streptavidin (Invitrogen) was used to incubate with cell lysates to pull down RNA complex. After RNA purification, RT-qPCR was carried out to determine the expression of premature miR-1182.

### 2.8 BrdU assay

Transfected cells were collected at 24, 48, and 72 h post-transfection and were transferred to a 6-well plate. Each well contained 10^5^ cells. Cell culture was carried out under the aforementioned conditions, followed by the addition of 10 µM BrdU (D Pharmingen). After incubation with BrdU for 2 h, cells were fixed, followed by incubation with anti-BrdU-antibody (peroxidase-coupled, Sigma-Aldrich) for 10 min. After that cells were washed and further incubated with peroxidase substrate for 1 h. Finally, optical density (OD) values were measured at 450 nm to reflect cell proliferation.

### 2.9 Western blot

Total proteins were isolated using RIPA and quantified using bicinchoninic acid, followed by denaturation and gel electrophoresis (SDS-PAGE). After gel transfer and blocking, polyvinylidene difluoride membranes were incubated with primary antibodies of ki67 (ab15580, Abcam) and PCNA (ab18197, Abcam) in cold room overnight. After that, membranes were further incubated with secondary antibody. Finally, enhanced chemiluminescence solution was used to develop signals and the MyECL imager was used to capture signal.

### 2.10 MTT assay

Cells were collected after transfection and seeded onto a 96-well plate (1 × 10^4^ cells per well). Cells were cultivated for 48 h, followed by the addition of 20 µL of MTT solution (5 g/L). Incubation was performed for 4 h. Then, 150 µL of DMSO was added and oscillation was performed. OD values at 490 nm were detected.

### 2.11 Colony formation assay

Cells were collected after transfection and digested with trypsin. Digested cells were transferred to 6-well plates (300 cells per well). Cells were then cultivated under the aforementioned conditions and colony formation was observed 3 weeks later.

### 2.12 Statistical analysis

SPSS 19.0 statistical software (IBM) was used to compare datasets and plot images. Datasets were compared by Students’ *t* test. *p* < 0.05 was statistically significant.

## 3 Results

### 3.1 The expression of FAM230B and miR-1182 in paired tissue samples

**Figure 1:**
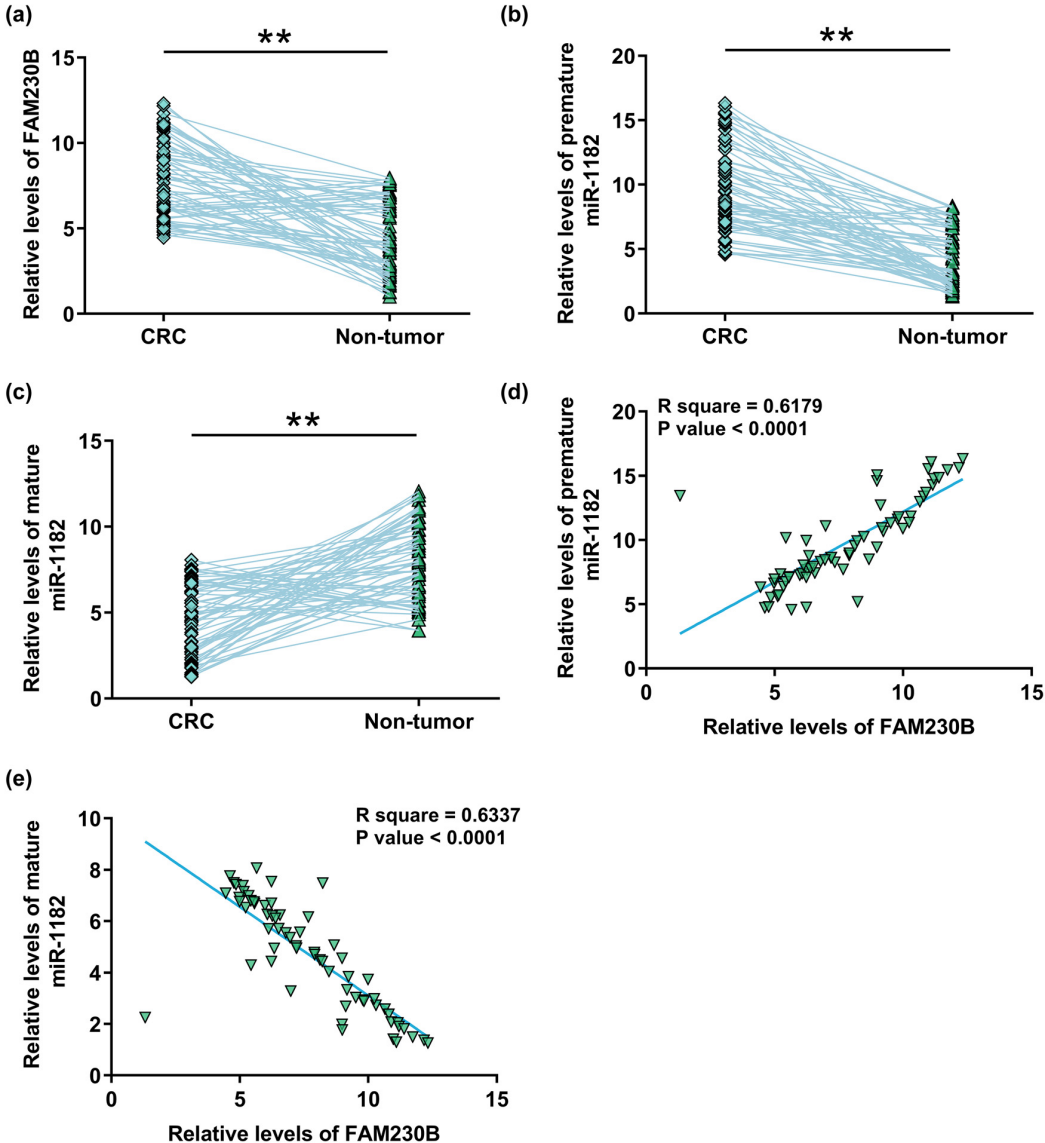
Analysis of the expression of FAM230B and miR-1182 in paired tissue samples. Paired CRC and non-tumor tissue samples from 64 CRC patients were used to extract total RNA samples, which were subjected to RT-qPCR to determine the expression of FAM230B (a), premature miR-1182 (b), and mature miR-1182 (c). The correlation between FAM230B and premature (d) or mature (e) miR-1182 was analyzed by Pearson’s correlation coefficient. ** *p* < 0.01.

The expression of FAM230B, premature miR-1182, and mature miR-1182 in CRC and paired non-tumor tissue samples from 64 CRC patients were determined using RT-qPCR. FAM230B (Figure 1a, *p* < 0.01) and premature miR-1182 (Figure 1b, *p* < 0.01) were highly upregulated in CRC tissues, while mature miR-1182 (Figure 1c, *p* < 0.01) was downregulated in CRC. Therefore, the upregulation of FAM230B and the suppressed maturation of miR-1182 might participate in CRC. The correlation between the expression of FAM230B and premature or mature miR-1182 across CRC tissues was analyzed by Pearson’s correlation coefficient. It showed that the expression of FAM230B was positively correlated with premature miR-1182 (Figure 1d), but inversely correlated with mature miR-1182 (Figure 1e).

### 3.2 The subcellular location of FAM230B and its interaction with miR-1182

**Figure 2:**
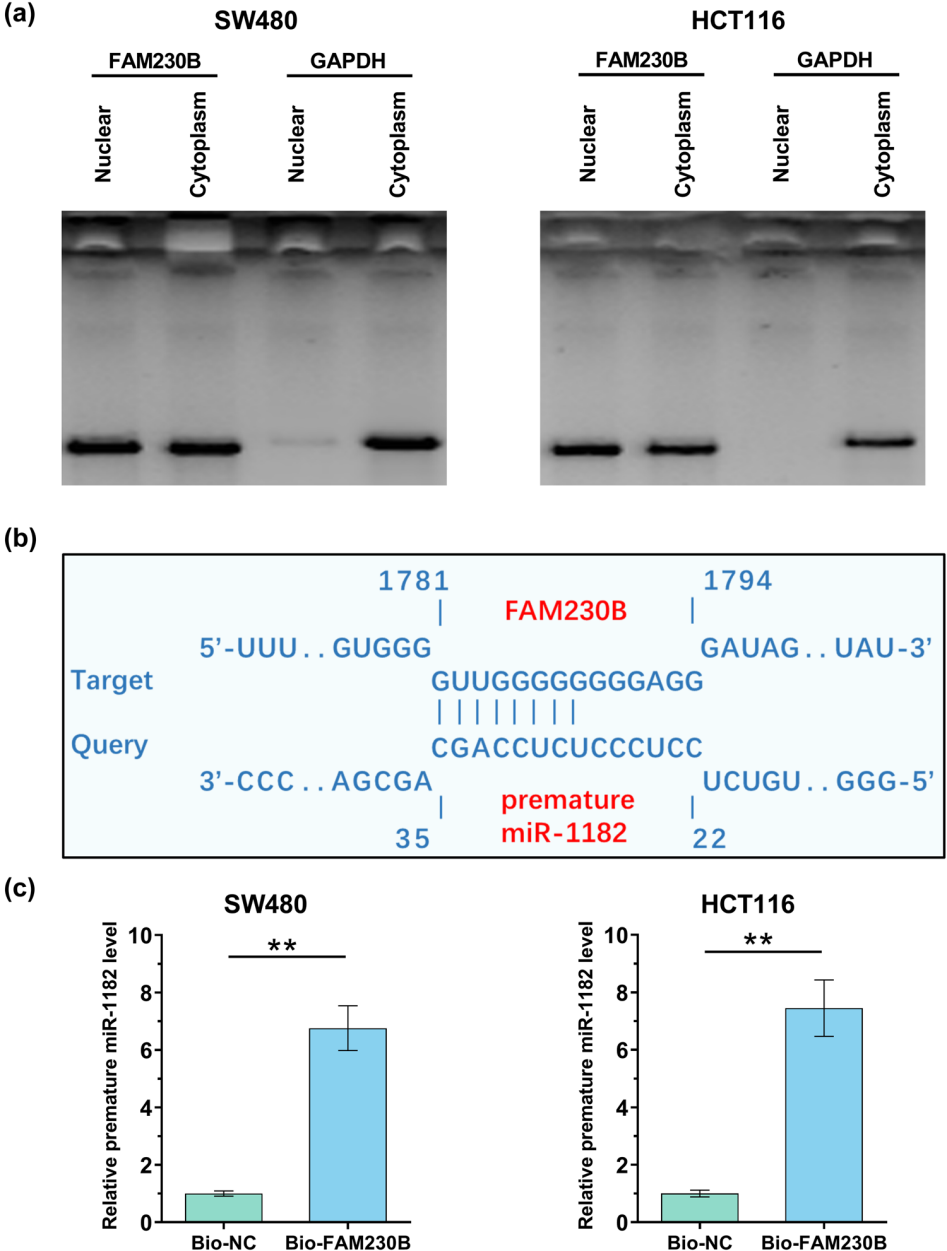
The subcellular location of FAM230B and its interaction with miR-1182. SW480 and HCT116 cells were subjected to the preparation of nucleus and cytoplasm samples, which were subjected to RT-qPCR to determine the expression of FAM230B (a). IntaRNA 2.0 was applied to predict that the base pairs could be formed by FAM230B and miR-1182 (b). The direct interaction between them was confirmed by RNA-RNA pull-down assay (c). ** *p* < 0.01.

SW480 and HCT116 cells were subjected to the preparation of nucleus and cytoplasm samples, which were subjected to RT-qPCR to determine the expression of FAM230B. It was observed that FAM230B could be detected in both nucleus and cytoplasm (Figure 2a). IntaRNA 2.0 was applied to predict the binding of miR-1182 to FAM230B. It was observed that FAM230B and miR-1182 could form strong base pairing (Figure 2b). The direct interaction between them was confirmed by RNA-RNA pull-down assay. Compared to Bio-NC pulldown group, Bio-FAM230B group showed significantly higher level of premature miR-1182, confirming the direct interaction between them (Figure 2c).

### 3.3 The involvement of FAM230B in the maturation of miR-1182

**Figure 3:**
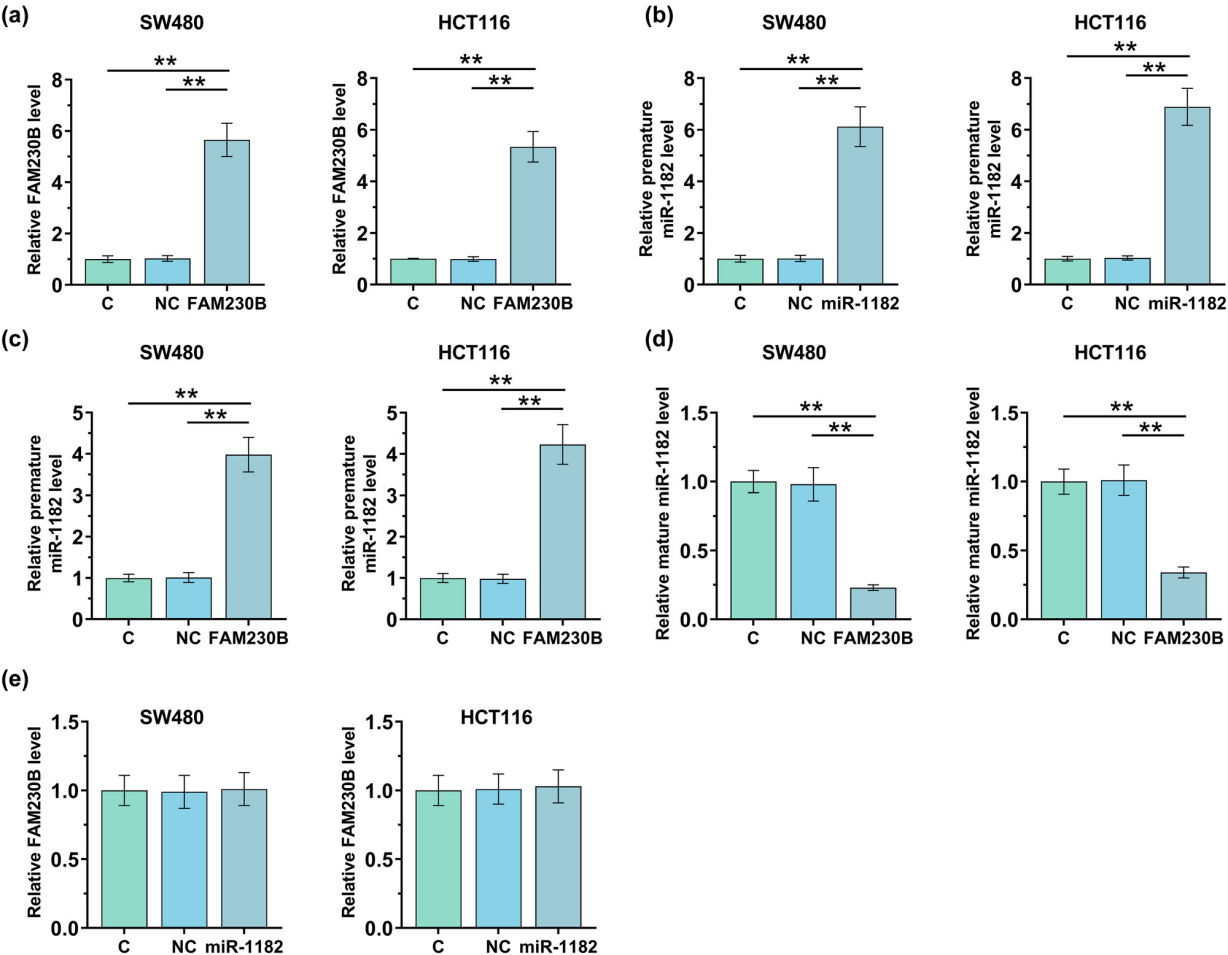
The involvement of FAM230B in the maturation of miR-1182. SW480 and HCT116 cells were overexpressed with FAM230B or miR-1182 by transfecting FAM230B expression vector or miR-1182 mimic. The overexpression of FAM230B (a) and miR-1182 (b) was confirmed by RT-qPCR at 48 h post-transfection. The expression of premature miR-1182 (c) and mature miR-1182 (d) in cells with FAM230B overexpression was determined by RT-qPCR. The expression of FAM230B in cells with miR-1182 overexpression was also analyzed with RT-qPCR (e). ** *p* < 0.01.

FAM230B or miR-1182 was overexpressed in SW480 and HCT116 cells by transfecting FAM230B expression vector or miR-1182 mimic. The overexpression of FAM230B (Figure 3a, *p* < 0.01) and miR-1182 (Figure 3b, *p* < 0.01) was confirmed by RT-qPCR at 48 h post-transfection. The expression of premature miR-1182 and mature miR-1182 in cells with the overexpression of FAM230B was determined by RT-qPCR. It was observed that overexpression of FAM230B increased the expression levels of premature miR-1182 (Figure 3c, *p* < 0.01) but decreased the expression levels of mature miR-1182 (Figure 3d, *p* < 0.01) in CRC cells. The expression of FAM230B in cells with the overexpression of miR-1182 was also analyzed with RT-qPCR. It was observed that overexpression of miR-1182 has no effect on the expression of FAM230B (Figure 3e).

### 3.4 The role of FAM230B and miR-1182 in the proliferation, viability, and colony formation of CRC cells

**Figure 4:**
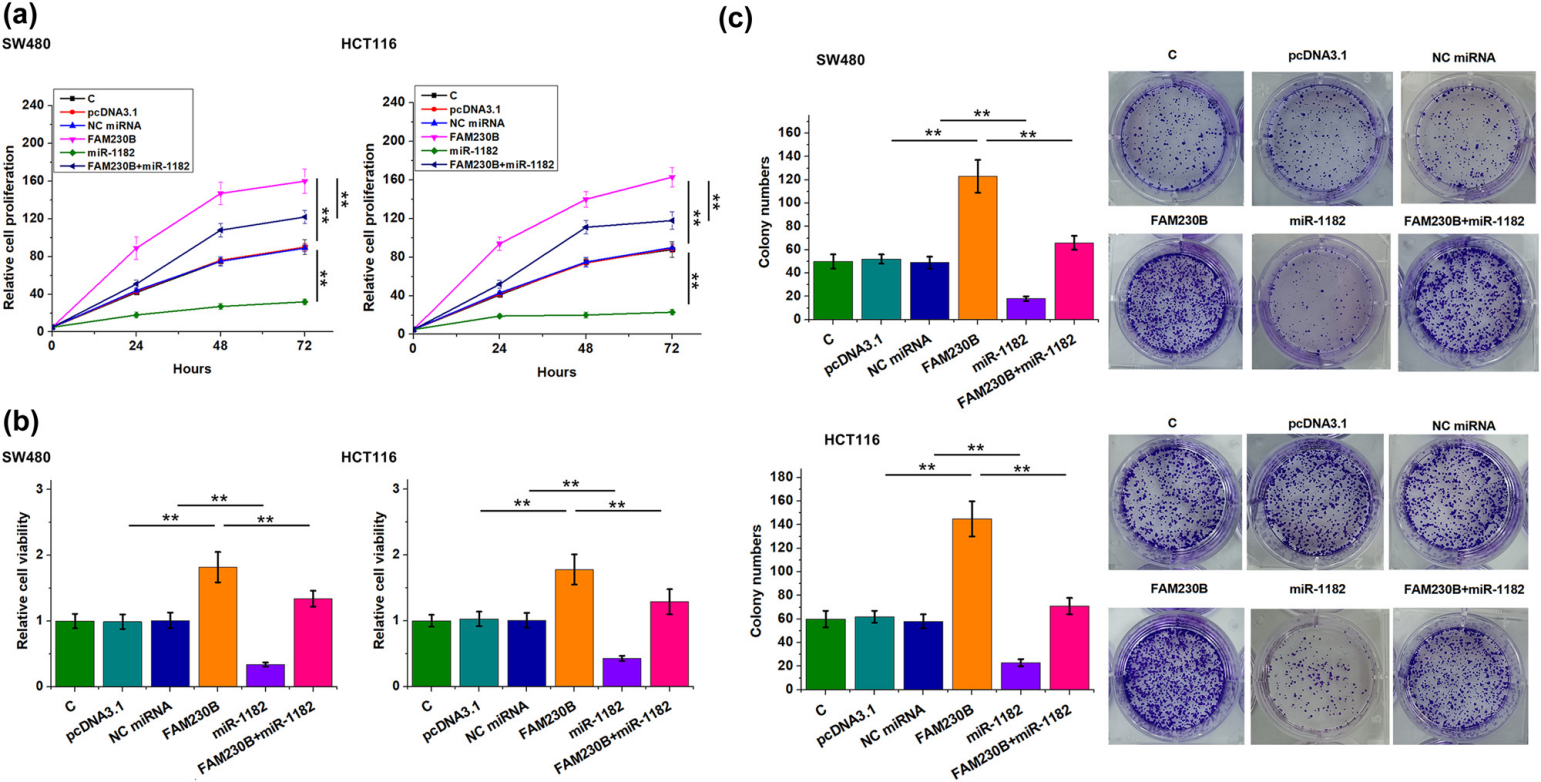
The role of FAM230B and miR-1182 in the proliferation of CRC cells. The role of FAM230B and miR-1182 in the proliferation, viability, and colony formation of SW480 and HCT116 cells was analyzed by BrdU assay (a), MTT assay (b), and colony formation assay (c), respectively. ** *p* < 0.01.

**Figure A1:**
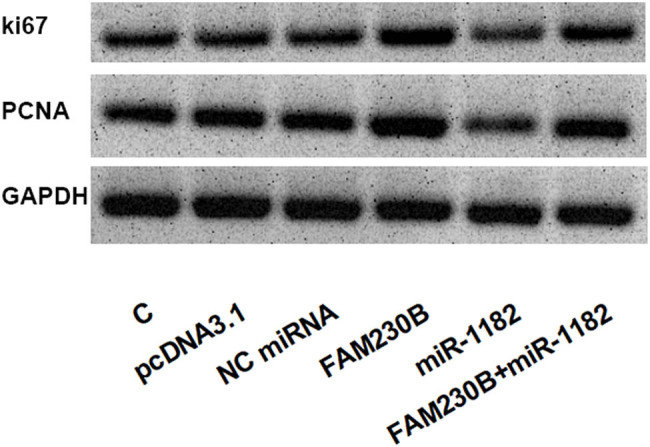
Expression of cell proliferation markers ki67 and PCNA.

The role of FAM230B and miR-1182 in regulating the proliferation, viability, and colony formation of SW480 and HCT116 cells was analyzed by BrdU assay (Figure 4a), MTT assay (Figure 4b), and colony formation assay (Figure 4c), respectively. FAM230B promoted CRC cell proliferation, increased cell viability, and promoted colony formation. In contrast, miR-1182 suppressed cell proliferation. Moreover, FAM230B suppressed the role of miR-1182 in inhibiting CRC cell proliferation, decreasing cell viability, and suppressing colony formation (Figure 4, *p* < 0.01). Original images of colony formation assay are presented in Supplemental File 1. Cell proliferation markers ki67 and PCNA are also determined in each group and consistent results are observed (Figure A1).

## 4 Discussion

The present study explored the involvement of FAM230B and miR-1182 in CRC and the interaction between them. We found that FAM230B was highly expressed in CRC and miR-1182 maturation is suppressed in CRC. In addition, FAM230B may suppress the maturation of miR-1182 to increase CRC cell proliferation.

The involvement of FAM230B in cancer biology has only been investigated in papillary thyroid cancer and gastric cancer [15,16]. FAM230B is upregulated in papillary thyroid cancer and increases the expression levels of WNT5A by sponging miR-378a-3p to accelerate tumor metastasis [15]. In another study, FAM230B was reported to be upregulated in gastric cancer and promotes tumor metastasis and growth by sponging miR-27a-5p to upregulate TOP2A [16]. However, the role of FAM230B in other cancers is unclear. We observed increased expression levels of FAM230B in CRC. In addition, overexpression of FAM230B increased CRC cell proliferation. Therefore, FAM230B plays an oncogenic role in CRC. However, *in vivo* animal model experiments are needed to validate the role of FAM230B in CRC tumor growth.

The tumor suppressive role of miR-1182 has been reported in several types of cancer [17,18]. In both bladder cancer and gastric cancer, miR-1182 targets hTERT to suppress tumor progression [17,18]. However, the involvement of miR-1182 in CRC is unclear. The present study showed that mature miR-1182 is downregulated in CRC and its overexpression resulted in decreased cell proliferation. However, premature miR-1182 is highly upregulated in CRC. Therefore, miR-1182 is likely a tumor suppressor in CRC, and the inhibited maturation, not the decreased transcription, is involved in CRC.

To date, the upstream regulators of miR-1182 in cancers are unclear. In the present study we showed that FAM230B could directly interact with premature miR-1182 and its overexpression decreased the maturation of miR-1182. In view of the fact that FAM230B can be detected in both nucleus and cytoplasm of CRC cells and premature miRNAs are only localized to nucleus, we speculated that FAM230B could sponge premature miR-1182 in nucleus to suppress its translocation to cytoplasm, thereby inhibiting its maturation.

In conclusion, FAM230B is upregulated in CRC, and the maturation of miR-1182 is suppressed in CRC. FAM230B may suppress the maturation of miR-1182 to increase CRC cell proliferation.
